# Mediterranean Diet and Its Correlates among Adolescents in Non-Mediterranean European Countries: A Population-Based Study

**DOI:** 10.3390/nu9020177

**Published:** 2017-02-22

**Authors:** Dario Novak, Lovro Štefan, Rebeka Prosoli, Arunas Emeljanovas, Brigita Mieziene, Ivana Milanović, Snežana Radisavljević-Janić

**Affiliations:** 1 Faculty of Kinesiology, University of Zagreb, Zagreb 10-000, Croatia; dario.novak@kif.hr (D.N.); rebeka.prosoli@kif.hr (R.P.); 2 Sports Education Faculty, Lithuanian Sports University, Kaunas 44221, Lithunia; arunas.emeljanovas@lsu.lt (A.E.); brigita.mieziene@lsu.lt (B.M.); 3 Faculty of Sport and Physical Education, Belgrade 11-000, Serbia; ivana.milanovic@fsfv.bg.ac.rs (I.M.); snezana.radisavljevic@fsfv.bg.ac.rs (S.R.-J.)

**Keywords:** nutrition, secondary-school students, health, physical activity, sedentary behavior

## Abstract

Little is known about the factors which might influence the adherence to a Mediterranean diet in non-Mediterranean European countries. Thus, the main purpose of this study was to determine the associations between socioeconomic, psychological, and physical factors on a Mediterranean diet. In this cross-sectional study, participants were 14–18-year-old adolescents (*N* = 3071) from two non-Mediterranean countries: Lithuania (*N* = 1863) and Serbia (*N* = 1208). The dependent variable was Mediterranean diet, and was assessed with the Mediterranean Diet Quality Index for children and adolescents questionnaire. Independent variables were gender, body-mass index, self-rated health, socioeconomic status, psychological distress, physical activity, and sedentary behavior. The associations between dependent and independent variables were analyzed by using logistic regression. Results showed that higher adherence to a Mediterranean diet was associated with higher self-rated health, socioeconomic status, and physical activity, yet low adherence to a Mediterranean diet was associated with being female, having higher body-mass index, psychological distress, and sedentary behavior. Our findings suggest that future studies need to explore associations between lifestyle habits—especially in target populations, such as primary and secondary school students.

## 1. Introduction

A Mediterranean diet is often characterized by a high consumption of fruits, vegetables, legumes, nuts, cereals, and olive oil, a low intake of saturated lipids, a moderately high intake of fish, dairy products, and ethanol (primarily in the form of wine), and a low intake of saturated lipids and sweets [1,2]. Several studies have shown that following the Mediterranean diet pyramid (consumption of grains, fruits, and vegetables, legumes, olive oil, low-fat cheese, and yogurt daily, fish and eggs weekly, and meat monthly)has a positive effect against cardiovascular [3,4], metabolic [5,6,7], and psychological health problems, and stroke [8]. Moreover, greater adherence to a Mediterranean diet was associated with a significant reduction in overall mortality, including mortality from cardiovascular diseases, cancer, and neuro-psychological diseases like Alzheimer’s or Parkinson’s disease [9].

Recently, low adherence to a Mediterranean diet has often been associated with obesity [10,11]. According to the World Health Organization (WHO) [10], in 2014, around 1.3 billion people aged 18 years or older were overweight, while 600 million were obese. In children and youth, the prevalence of overweight and obesity has increased in the past several years [12,13], causing premature deaths and increasing the risk for cardiovascular and metabolic diseases [10]. It has also been established that childhood obesity was associated with a higher chance of obesity and disability in adulthood [10,14]. Another great public health problem which mainly effects children and youth is physical inactivity [9]. The highest prevalence of physical inactivity in 2008 was in American and Eastern Mediterranean adolescents [10]. A few studies have demonstrated that children and youth with unhealthy life habits—such as a sedentary lifestyle and poor nutrition—represent the target group for specific interventions [15].

Several studies have explored the associations between the factors associated with a Mediterranean diet, especially in southern Mediterranean countries and in different populations of older adults [16], the general population [17], and adolescents [11,18,19,20]. Findings from these studies suggested that physical activity [11,19] and socioeconomic status [11,21] were associated with higher adherence to a Mediterranean diet, yet body-mass index [11] and sedentary behavior [22] were associated with lower adherence to a Mediterranean diet. 

Regions seem to play a role as well; one study showed that Italy had the highest adherence to a Mediterranean diet, followed by Central and Northern European countries, while children in Spain and Cyprus showed the lowest adherence [23]. Clearly, findings have shown that Mediterranean countries do not always have the highest adherence to a Mediterranean diet, although they are geographically situated in the Mediterranean region of Europe. Due to these findings, it was necessary to investigate how specific factors could influence adherence to a Mediterranean diet in non-Mediterranean countries. Thus, the main purpose of this study was to determine the associations between a Mediterranean diet and its potential correlates in two non-Mediterranean European countries among adolescents. We hypothesized that recommended physical activity levels, higher parental socioeconomic status, and higher self-rated health would be associated with higher adherence to a Mediterranean diet, yet body-mass index, psychological distress, and sedentary behavior would be inversely associated with higher adherence to a Mediterranean diet. Gender is included as a control variable.

## 2. Materials and Methods

### 2.1. Participants and Data Collection 

This cross-sectional study included 3200 adolescents 14–18 years of age from 10 and 15 randomly selected secondary-schools in Lithuania and Serbia, respectively. Randomization of schools was done with replacement by drawing school codes on slips of paper from a box, with each school having equal probability of selection. Specifically, school selection was done according to school districts in both Lithuania and Serbia. Before the study began, all participants gave assent, and their parents/guardians had given informed consent to participate in the study. From all participants enrolled in the study, 29 chose not to participate, while 100 returned the questionnaires with incomplete data. At the end, a total of 3071 participants were enrolled in the study. All the procedures involved in this study were approved by the Institiutional Review Board of the Faculty of Kinesiology, University of Zagreb, Croatia.

Data were collected between January and March in the 2015–2016 school year performed by five trained doctors. The procedure was consistent for all participants. Participants completed the questionnaires during class hours in the presence of researchers. At the beginning of the class, researchers explained the main purpose of the study and the study protocol. After the explanation, students completed the questionnaires and put them inside a box provided. The researchers were available to explain or clarify the questions to the participants. The procedure took about 30 min and was anonymous.

The Mediterranean Diet Quality Index for children and adolescents questionnaire was used to evaluate the adherence to a Mediterranean diet in adolescents. The questionnaire consists of 16 items, where there are four questions denoting a negative connotation to the Mediterranean diet (consumption of fast food, baked goods, sweets, and skipping breakfast) and 12 questions denoting positive connotation we confirm that our intended meaning is retained (oil, fish, fruits, vegetables, cereals, nuts, pulses, pasta or rice, dairy products and yoghurt consumption). Questions denoting negative connotation are scored with −1, while positive connotation questions are scored with +1. According to the KIDMED index, a score of 0–3 reflects a poor adherence to the Mediterranean diet, a score of 4–7 describes average adherence, and a score of 8–12 shows a good adherence [24]. For the purpose of this study, we created two groups, such that participants who scored ≤3 were collapsed into one category (poor adherence to the Mediterranean diet), while average and good adherence were designed as the second category (good adherence to the Mediterranean diet) [11].

### 2.2. Correlates

Self-rated health was assessed by using one item: “How would you perceive your health?” with five possible answers: (1) very poor; (2) poor; (3) fair; (4) good; and (5) excellent [25]. We binarised responses, where responses “very poor” and “poor” collapsed into “poor health”, while responses “fair”, “good”, and “excellent” represented “good health”. Previous studies have showed that self-rated health served as a reliable predictor of mortality, especially in children and adolescents [26]. Specifically, poor self-rated health was associated with increased mortality during 27 years of follow-up. Additionally, alcohol and-drug-related mortality, along with the three psychological factors (low emotional control, psychiatric diagnosis, and self-reported medication for nervous problems) were all associated with the increased mortality in the group which reported having poor or very poor self-rated health [26].

As a measure of physical activity in the last 7 days, we used the International Physical Activity Questionnaire-short form [27]. The results were expressed in metabolic-equivalent hours per week. Participants were categorized as highly active (60 min or more on all 7 days), medium active (30–59 min on all 7 days) and low active (<30 min on all 7 days) [28].The International Physical Activity Questionnaire has previously been shown to have adequate reliability and validity [27]. 

As additional correlates, we used body-mass index (BMI) from self-reported height and weight of the participants. The international age- and gender-specific child BMI cut-off points for children developed by the Childhood Obesity Working Group of the International Obesity Task Force were used to define subjects as normal-weight, overweight, or obese [28].To assess socioeconomic status, we used both parents’ occupational status at the time the study was conducted. The questionnaire consisted of six questions (questions 1and 2denoted high socioeconomic status, like managers and professionals; 3–4 white collar; and 5–6 blue collar) [29]. It is worth mentioning that all participants had both biological parents available for fulfilling the questionnaire. Additionally, when the mother’s occupational status was discordant from the father’s, in this case, we used father’s status, as in both analyzed countries, fathers are usually primary income earners for their families, and family socio-economic level depend mostly on them.

To assess psychological distress, we used the six-item Kessler scale: (1) “How often during the past month did you feel nervous?”; (2) “How often during the last month did you feel hopeless?”; (3) “How often during the last month did you feel restless or fidgety?”; (4) “How often during the last month did you feel so depressed that nothing could cheer you up?”; (5) “How often during the last month did you feel that everything was an effort?”; and (6) “How often during the last month did you feel worthless?”. Each question was scored from 0 to 4 (none of the time–all of the time). Scores from all six questions were summed up (range 0–24). Lower scores indicated a lower level of psychological distress. We dichotomized the results, where scores between 0–12 represented participants without psychological distress, and scores ≥13 represented participants with psychological distress [30]. This questionnaire showed good reliability and validity in adolescents [31].

Sedentary behavior was assessed a by singleitem: “In the last 7 days, how much time did you spend sitting on a week day?” The responses were expressed as minutes per day. Responses were categorized as ≤120 min/day or >120 min/day [32].

### 2.3. Data Analysis

Categorical variables are presented as frequencies and percentages. Differences between gender in categorical variables were tested by using Chi-square test. The associations between dependent (low vs. average/good adherence) and independent variables were analyzed by using logistic regression analysis with odds ratios (ORs) and 95% confidence intervals (95% CIs). Furthermore, the cluster effect was considered in the analysis to avoid the possibility that the measurements within school might not be independent. For this, we adjusted the standard errors (SEs) by computing clustered robust SEs for the coefficients. We calculated the associations between the dependent and each independent variable entered separately into the model (seven models). We also calculated the associations between dependent and all seven independent variables when entered simultaneously into the model. All the analyses were performed by using SPSS ver. 22 (Statistical Package for Social Sciences, Chicago, IL, USA). Statistical significance was set up at *p* ≤ 0.05, and were based on two-sided tests.

## 3. Results

Basic descriptive statistics of the study participants are presented in Table 1. In total, 63% of male and 58% of female adolescents had average/good adherence to a Mediterranean diet. Male students had higher values of body-mass index, due to their morphological characteristics, but were also significantly more physically active than females. A higher percentage of female students experienced psychological distress in the past month than male students (*p* < 0.001). Moreover, around 70% of both male and female students were sedentary for at least 2 h per day.

The associations between dependent and independent variables are presented in Table 2. When entered individually into the model, average/good adherence to a Mediterranean diet was associated with good self-rated health (OR 1.87; 95% CI 1.39 to 2.51), medium (OR 1.81; 95% CI 1.49 to 2.19), and highphysical activity (OR 2.07; 95% CI 1.75 to 2.46). Poor adherence to a Mediterranean diet was associated with being a female student, (OR 0.75; 95% CI 0.65 to 0.86), being overweight/obese (OR0.76; 95% CI 0.61 to 0.93), having a higher psychological distress (OR 0.64; 95% CI 0.52 to 0.78), spending >120 min/day in sitting position (OR 0.75; 95% CI 0.61 to 0.94), and having low socioeconomic status (OR 0.70; 95% CI 0.59 to 0.82). When all the variables were entered simultaneously into the model (model eight), the same associations between Mediterranean diet and its correlates remained significant.

## 4. Discussion

The main purpose of the present study was to determine the associations between the adherence to a Mediterranean diet and gender, body-mass index, self-rated health, socioeconomic status, psychological distress, moderate-to-vigorous physical activity, and sedentary behavior among adolescents in the non-Mediterranean European countries Lithuania and Serbia.

The prevalence of low adherence to a Mediterranean diet in our study was 39.0%.This is higher compared to other studies in the Mediterranean region [17,19,24,33], yet the percentage of adolescents who reported average adherence to Mediterranean diet in our study was similar to other Mediterranean populations [19,24]. As mentioned before, it is generally believed that Mediterranean countries have higher adherence to Mediterranean diet compared to other non-Mediterranean countries [23]. However, previous findings have shown that Mediterranean populations are gradually moving away from the traditional Mediterranean diet [34], due to a stressful lifestyle and less time to spend in the kitchen cooking [35].For example, more than 13% of students in our study had good adherence to Mediterranean diet, while the proportion of well-adhered students in Southern Italy were slightly lower; i.e., about 9% [1]

Results from our study showed that female students were less likely to have higher adherence to a Mediterranean diet than male students, which is inconsistent with past studies [19,36]. The above-mentioned differences between our study and previous findings could be explained by two possible reasons. First, the prevalence of meeting the recommendations of moderate-to-vigorous physical activity was higher among male students. Few studies showed positive associations between physical activity and higher adherence to a Mediterranean diet [11,19]. Second, higher physical activity levels in male students may causeless time spent in sedentary behavior compared to female students. One study showed that female students with poor adherence to a Mediterranean diet were three times more likely to participate in sedentary behavior, and had lower adherence to a Mediterranean diet [22]. Moreover, the same study showed that the use of media-screen and homework did not affect participation in physical activity in male students, yet were enough to distinguish active from sedentary female students [22]. Additionally, female students tend to have higher academic achievement than male students, pointing out that they spend more time doing their homework, and in general, completing their obligations toward school [37]. Since sedentary behavior is associated with higher academic achievement [37], yet with poorer adherence to a Mediterranean diet [22], it is possible that female students spend more time sitting due to their school obligations, often accompanied by stress and irregular eating, which in addition leads to poorer adherence to Mediterranean diet.

Our findings showed an inverse association between body-mass index and adherence to a Mediterranean diet, which is in accordance with past studies [11,19,38,39,40]. In one recent clinical trial conducted among adolescents in Mexico, results showed that a Mediterranean diet reduced body-mass index in the intervention group compared to a standard diet group [39]. Moreover, in another recent study, findings showed that body-mass index was inversely associated with a Mediterranean diet, but through higher levels of physical activity and lower levels of sedentary behavior reported by children with higher adherence to a Mediterranean diet [40]. Through additional analysis, our study showed the same mechanisms, where participants who reported higher adherence to a Mediterranean diet were more physically active and had lower sedentary time compared to their peers with lower adherence to a Mediterranean diet.

Our results showed an association between good self-rated health and higher adherence to a Mediterranean diet, which is in accordance with some previous studies [41,42]. Previous findings showed that higher adherence to a Mediterranean diet had positive and beneficial effects on both physical and mental health components [41]. This was attributed to antioxidants, which are generally present inside Mediterranean food. Antioxidants can be found in vitamins (specifically vitamins A, C, and E), but also in a number of compounds capable of protecting against oxidative damage [43]. Some of these compounds are known as phytochemicals, such as cistein (found in garlic), brassin (found in broccoli and kale), flavones (found in citrus fruits), polyphenols (found in green teas), and oleuropein (found in olive oil), which have anti-inflammatory effects against cardiovascular [3], metabolic [6], and psychological [8] health problems. Another study from Spain showed significant direct associations between adherence to a Mediterranean diet and general health [42]. Findings from the Spanish study revealed that those participants who had improved their initial Mediterranean diet scores had better scores in physical and mental components and general health [38]. Adherence to a Mediterranean diet showed beneficial effects during childhood on symptoms of asthma and rhinitis. Results from that study showed that diet might explain the relative lack of allergic symptoms in this population [43].

Next, our findings revealed the association between higher adherence to a Mediterranean diet and middle/high socioeconomic status. Previous findings showed the same associations [21,22,44,45]. More specifically, one study concluded that more-educated people often consume high-quality and nutritional-rich products, as opposed to lower socioeconomic groups, who have a higher risk to develop nutrition-associated diseases [45]. Another potential factor influencing lower adherence to a Mediterranean diet in low socioeconomic groups is money. To clarify, one study showed that Mediterranean products cost more than Western products, so groups with low monthly income cannot afford such foods, and often reach for a nutrient-poor diet [46]. 

Psychological distress was inversely associated with adherence to a Mediterranean diet in our study. One recent study showed that nutrient rich foods with folate, zinc, and magnesium were inversely associated with depressive symptoms [47], while food enriched with long-chain omega-3 fatty acids were inversely associated with anxiety problems [48]. In general, one recent meta-analysis showed that high adherence to a Mediterranean diet was associated with reduced risk of depression and cognitive impairment. Additionally, participants who reported having average adherence to a Mediterranean diet had similar associations with reduced risk for depression and cognitive impairment, independent of age [8]. Several studies have shown that a Mediterranean diet has positive effects against cardiovascular diseases, such as hypertension [49], coronary heart disease [49], and dyslipidemia [49], which are risk factors for Alzheimer’s disease. Another possible mechanism linking a Mediterranean diet and Alzheimer’s disease are the antioxidant properties of Mediterranean products, such as olive oil, vitamins, and carotenoids, which may reduce markers of oxidative stress and lower risk for Alzheimer’s disease [50].

A higher level of physical activity was associated with higher adherence to a Mediterranean diet in our study. Previous findings showed the same associations in Mediterranean adolescents [11,19,22,51]. It is possible that healthy lifestyle—such as higher levels of physical activity, lower levels of sedentary time, and higher adherence to a Mediterranean diet—tend to cluster and form aso-called “Mediterranean lifestyle”. Here, physical activity may serve as a protective factor against different diseases [17,18]. Additionally, results from our study showed that more sedentary adolescents were less likely to have high adherence to Mediterranean diet as opposed to their non-sedentary peers, which is in accordance with some previous studies [22,52]. A study by Bibiloni et al. [22] specifically showed that sedentary adolescents often consume less cereals and fresh fruits, while high fat foods were often in their diet. Moreover, one study showed that children who spent ≥4 h per day on a media-screen showed lower mean intake of yogurt, fruits, and vegetables, yet increased mean intake of the “Western” dietary patterns (i.e., sweets, chocolates, and food rich with saturated fatty acids) [52]. Additionally, a recent study from Peng et al. [53] showed that having poor adherence to a Mediterranean diet was higher in those adolescents who watched television/videos/listened to music ≥2 h/day and who did not participate in aerobic activities or ball games weekly.

Our study has several limitations. First, due to the cross-sectional design, we cannot exclude the possibility of reverse causality, meaning that higher adherence to a Mediterranean diet led to good self-rated health, higher levels of physical activity, or socioeconomic status, yet lower levels of body-mass index, psychological distress, and sedentary behavior. Second, we used a subjective measurement to assess adherence to a Mediterranean diet and its correlates. Specifically, to assess physical activity and sedentary behavior, we used the International Physical Activity Questionnaire-short form. Although this questionnaire showed satisfactory reliability and validity [27], the use of subjective measures often leads to overestimation [54]. Moreover, we used a self-reported measure for height and weight. Previous studies showed that both male and especially female adolescents tended to underestimate their body-mass index [54]. Third, sedentary behaviors may be more difficult to remember, and we cannot exclude recall bias in self-reported sedentary behavior in adolescents [55]. Fourth, we did not ask the participants about their cigarette and alcohol consumption. This is a limitation, since previous studies have shown that these two factors may influence adherence to a Mediterranean diet [19].

## 5. Conclusions

This study reports important information about non-Mediterranean European adolescents. Our results agree with past research conducted in Mediterranean settings, showing for example a socioeconomic gradient in the adherence to the Mediterranean diet both in adolescents and adult individuals [1,21]. Findings from our study show that by categories, female students, overweight, and obese adolescents who do not meet the recommendations for participating in physical activity and are sedentary are less likely to have higher adherence to a Mediterranean diet. These adolescents represent at-risk groups, and future studies should track lifestyle behaviors and implement specific strategies and policies within the family, neighborhood, and school system to increase the positive trend for healthy life habits (i.e., home-cooked meals, leisure time, organized physical activity, and less time spent in sedentary behaviors).

## Figures and Tables

**Table 1:** Basic descriptive characteristics of the study participants.

Study Variables	Total (*N* = 3071)	Male Students (*N* = 1449)	Female Students (*N* = 1622)	*p* *
	*N* (%)	*N* (%)	*N* (%)	
Mediterranean Diet Quality Indexscore				
Poor adherence (≤3 points)	1195 (39.0)	519 (36.0)	676 (41.7)	
Average adherence (4–7 points)	1462 (47.7)	704 (48.8)	758 (46.8)	
Good adherence (≥8–12 points)	414 (13.3)	226 (15.2)	188 (11.5)	<0.001
Body-mass index				
Normal	2671 (86.6)	1184 (81.1)	1486 (91.6)	
Overweight/obesity	412 (13.4)	276 (18.9)	136 (8.4)	<0.001
Self-rated health				
Poor	180 (5.9)	74 (5.1)	106 (6.5)	
Good	2891 (94.1)	1375 (94.9)	1516 (93.5)	0.106
Socioeconomic status				
Low	382 (12.4)	170 (44.5)	212 (55.5)	
Middle	906 (29.8)	428 (46.6)	478 (53.4)	
High	1783 (57.8)	862 (48.3)	921 (51.7)	0.114
Psychological distress				
Low	2599 (84.6)	1297 (89.5)	1302 (80.3)	
High	472 (15.4)	152 (10.5)	320 (19.7)	<0.001
Moderate-to-vigorous physical activity				
Low	1511 (49.0)	567 (37.5)	944 (62.5)	
Medium	628 (20.4)	312 (49.7)	316 (50.3)	
High	932 (30.6)	581 (39.8)	363 (38.5)	<0.001
Sedentary behavior				
≤120 min/day	886 (28.9)	430 (29.7)	456 (28.1)	
>120 min/day	2185 (71.1)	1019 (70.3)	1166 (71.9)	0.339

* Chi-square test, *p* ≤ 0.05.

**Table 2:** Odds ratios (ORs) for average/good adherence to a Mediterranean diet.

Study Variables	Model 1	Model 2	Model 3	Model 4	Model 5	Model 6	Model 7	Model 8
	OR (95% confidence interval)	OR (95% confidence interval)	OR (95% confidence interval)	OR (95% confidence interval)	OR (95% confidence interval)	OR (95% confidence interval)	OR (95% confidence interval)	OR (95% confidence interval)
Gender								
Male students								
Female students	0.75 (0.65 to 0.86) ***							0.84 (0.72 to 0.99) *
Body-mass index								
Normal								
Overweight/obesity		0.76 (0.61 to 0.93) **						0.74 (0.59 to 0.92) **
Self-rated health								
Poor								
Good			1.87 (1.39 to 2.51) ***					1.70 (1.25 to 2.31) ***
Socioeconomic status								
High								
Middle				1.11 (0.89 to 1.40)				1.19 (0.94 to 1.49)
Low				0.70 (0.59 to 0.82) ***				0.75 (0.64 to 0.88) ***
Psychological distress								
Low								
High					0.64 (0.52 to 0.78) ***			0.73 (0.59 to 0.89) ***
Moderate-to-vigorous physical activity								
Low								
Medium						1.81 (1.49 to 2.19) ***		1.76 (1.45 to 2.15) ***
High						2.07 (1.75 to 2.46) ***		1.93 (1.61 to 2.30 ***
Sedentary behavior								
≤120 min/day								
>120 min/day							0.75 (0.61 to 0.94) **	0.76 (0.64 to 0.90) ***

Each regression model contains Mediterranean Diet Quality Index diet score and one other variable, except model eight, which includes all independent variables entered simultaneously.* *p* ≤ 0.05; ** *p* ≤ 0.01; *** *p* ≤ 0.001.
